# Gingipains from *Porphyromonas gingivalis* play a significant role in induction and regulation of CXCL8 in THP-1 cells

**DOI:** 10.1186/1471-2180-14-193

**Published:** 2014-07-18

**Authors:** Kartheyaene Jayaprakash, Hazem Khalaf, Torbjörn Bengtsson

**Affiliations:** 1 Faculty of Medicine and Health, Örebro University, Örebro, Sweden; 2 School Of Health and Medical Sciences, Örebro University, SE-701 82 Örebro, Sweden

**Keywords:** *Porphyromonas gingivalis*, THP-1 cells, Gingipains, Mutants, CXCL8 degradation

## Abstract

**Background:**

*Porphyromonas gingivalis* is an important bacterial etiological agent involved in periodontitis. The bacterium expresses two kinds of cysteine proteases called gingipains: arginine gingipains (RgpA/B) and lysine gingipain (Kgp). This study evaluated the interaction between *P. gingivalis* and THP-1 cells, a widely used monocytic cell line, *in vitro* with a focus on CXCL8 at the gene and protein levels and its fate thereafter in cell culture supernatants. THP-1 cells were stimulated with viable and heat-killed wild-type strains ATCC 33277 or W50 or viable isogenic gingipain mutants of W50, E8 (Rgp mutant) or K1A (Kgp mutant), for 24 hours.

**Results:**

ELISA and qPCR results show an elevated CXCL8 expression and secretion in THP-1 cells in response to *P. gingivalis*, where the heat-killed ATCC33277 and W50 induced higher levels of CXCL8 in comparison to their viable counterparts. Furthermore, the Kgp-deficient mutant K1A caused a higher CXCL8 response compared to the Rgp-deficient E8. Chromogenic quantification of lipopolysaccharide (LPS) in supernatant showed no significant differences between viable and heat killed bacteria except that W50 shed highest levels of LPS. The wild-type strains secreted relatively more Rgp during the co-culture with THP-1 cells. The CXCL8 degradation assay of filter-sterilized supernatant from heat-killed W50 treated cells showed that Rgp was most efficient at CXCL8 hydrolysis. Of all tested *P. gingivalis* strains, adhesion and internalization in THP-1 cells was least conspicuous by Rgp-deficient *P. gingivalis* (E8), as demonstrated by confocal imaging.

**Conclusions:**

W50 and its Kgp mutant K1A exhibit a higher immunogenic and proteolytic function in comparison to the Rgp mutant E8. Since K1A differs from E8 in the expression of Rgp, it is rational to conclude that Rgp contributes to immunomodulation in a more dynamic manner in comparison to Kgp. Also, W50 is a more virulent strain when compared to the laboratory strain ATCC33277.

## Background

Periodontitis is a disease affecting the supporting hard and soft tissues of the teeth characterized by loosening and eventual tooth loss [1]. Mounting epidemiological evidence has linked periodontitis to systemic illness such as atherosclerosis, and rheumatoid arthritis increasing the morbidity associated with the disease [2,3]. *Porphyromonas gingivalis* (*P. gingivalis)* is a natural member of the oral microflora but can be detected in great numbers in at least 85% of the periodontal lesions. There is a paradigm shift of the microbiome from health to disease and *P. gingivalis* has been identified as one of the key anaerobic proteolytic species instrumental in periodontal disease progression [4,5]. *P. gingivalis* is a gram negative, assacharolytic, black pigmented bacterium armed with a pleothera of virulence factors such as lipopolysaccharide (LPS), gingipains, peptidyl arginine deiminase, haemagglutinins, fimbriae and outer membrane proteins. These factors are indispensable for the persistence of the organism by enhancing biofilm formation and evading host defense mechanisms [6]. Gingipains are typsin-like cysteine proteases that are broadly classified into two main categories – (i) arginine gingipains (RgpA and RgpB) and (ii) lysine gingipain (Kgp), which can exist in soluble and membrane-bound forms [7].

Monocytes and neutrophils are sentinel cells of innate immunity and are found in abundance during periodontal infection [8]. THP-1 cells have been widely accepted and used as a surrogate for primary monocytes in biomedical research [9,10]. Toll-like recptors (TLRs) are germline encoded pattern recognition receptors (PRRs) present on various cells and they have been evolved to recognize conserved products unique to microbial metabolism and alert the immune system along signaling cascades. However, in chronic infections like periodontitis, a large number of these pathways converge on a relatively limited set of interaction mechanisms. Polymorphisms of the TLRs have been implicated in various diseases and susceptibility to infections [11,12]. Monocytes have various protease-activated receptors (PARs) that are activated by gingipains and other components of the bacteria. The PARs are a unique category of trans-membrane receptors that are activated on the cleavage of the receptor N-terminal part to expose a new, previously cryptic sequence. The exposed sequence remains tethered to the receptor and acts further as a receptor-activating ligand which results in Ca2+ increase and production of CXCL8 [13]. It has been shown that platelet activation with gingipains is associated with PARs and PAR1 and 4 are specifically involved in response to Rgp [14,15].

CXCL8 is an important signaling chemokine which is secreted in copious amounts by monocytes in response to infection and it serves to recruit neutrophils to the site of infection along a chemotactic gradient. Stathopolou and colleagues have studied modification of host cytokine responses by *P. gingivalis* using human gingival epithelial cells [16]. It has been documented that CXCL8 exists in two forms: (i) 72 aminoacid CXCL8 secreted by monocytes and lymphocytes and (ii) 77 aminoacid CXCL8 secreted by various cells of non-immunological origin. The latter form is broken down into a more potent truncated action, but on prolonged exposure, they are completely inactivated [17].

Gingipains have also been known stimulate an innate immune response followed by a potent down regulation of its effects by proteolytic degradation of complement components, anti-bacterial peptides, cytokines and chemokines thereby preventing the resolution of the infection [18,19]. Several studies documenting the effect of purified gingipains or specific gingipain mutants on cells and secretory proteins from the cells have been carried out so far. Previous studies have demonstrated cytokine and chemokine production in THP-1 cells and various other cell lines when stimulated with the whole bacterium or components of *P. gingivalis *[16,20-22]. However, the mechanisms involved are still poorly understood.

We hypothesize that, arginine and lysine gingipains differentially regulate CXCL8 expression and secretion in monocytes. The aim of this study is to clarify the role of gingipains in the expression, release and degradation of CXCL8 in *P. gingivalis*- stimulated THP-1 cells by using specific Kgp or Rgp mutants.

## Methods

### Cell culture

THP-1 (TIB-202, American Type culture collection, Manassas, VA, USA) cells were grown in RPMI-1640 containing 10% fetal bovine serum at 37°C, 5% CO_2_. The logarithmic growth of the cells was maintained between 2 × 10^5^ to 1 × 10^6^ cells/ml by passage, every 3–4 days. A cell concentration of 1 × 10^6^ cells /ml per well was used in a six well plate during each experiment.

### Bacterial culture and preparation

*Porphyromonas gingivalis* ATCC 33277(American Type Culture Collection, Manassas, VA, USA), W50 and its isogenic mutant strains: arginine gingipain (Rgp) mutant strain E8 and lysine gingipain (Kgp) mutant strain K1A were kind gifts from Dr. M. Curtis (Barts and The London, Queen Mary's School of Medicine and Dentistry, UK), were grown in fastidious anaerobic broth (Lab M Limited, Lancashire, UK). The bacteria were cultured for 72 hours in an anaerobic chamber (Concept 400 Anaerobic Workstation; Ruskinn Technology Ltd., Leeds, UK) containing 80% N2, 10% CO2, and 10% H2 at 37°C.

The bacteria was centrifuged at 9300 × g for 10 minutes at room temperature, washed twice with Krebs-Ringer glucose buffer (KRG) free of calcium (120 mM NaCl, 4.9 mM KCl, 1.2 mM MgSO4, 1.7 mM KH2PO4, 8.3 mM Na2HPO4, and 10 mM glucose, pH 7.3) and resuspended in fresh KRG buffer without calcium and the concentration was adjusted to 1 × 10^9^ CFU/ml. thirty microlitres of each serial dilution suspensions were plated on fastidious anaerobic agar (Acumedia, Neogen, Lansing, USA) plate enriched with 5% defibrinated horse blood and incubated for 7 days in the anaerobic chamber for a subsequent colony count. For heat-killing the bacteria, the prepared bacteria was placed in 70°C heating block for one hour. To ensure heat-killing, 10μl of this bacterial suspension was streaked on to a fastidious anaerobic agar plate plate enriched with 5% defibrinated horse blood and incubated for 7 days in the anaerobic chamber with no observed growth. In order to stimulate the THP-1 cells, a MOI (multiplicity of infection) 100 bacteria was used and incubated for 24 hours in a stable environment of 37°C, 5% CO_2_ and 95% air.

### Enzyme-linked immunosorbant assay (ELISA)

ELISA was performed on culture supernatant of THP-1 cells challenged with the different strains of *P. gingivalis* to measure CXCL8. After the 24 hour treatment, the cell culture suspension was centrifuged at 1200 × g for 5 minutes at room temperature and the cell - free supernatants were aliquoted and stored at -80°C until further analysis using Human CXCL8 ELISA kit (Biolegend, Sandiego, USA).

### Reverse transcriptase quantitative real-time PCR (RT- qPCR)

The *CXCL8* expression of THP-1 cells in response to the various strains of *P. gingivalis* was measured using RT-qPCR. RNA isolation was carried out using Genejet RNA isolation kit (Fermentas, Sweden) according to manufacturer’s protocol. Reverse transcription was done with equal amounts of RNA using cDNA synthesis kit (BioRad, Sweden). The primer sequences for *CXCL8* are as follows: forward sequence: GAGAGTGATTGAGA GTGGACCAC; reverse sequence: CACAACCCTCTGCACCCAGTTT (product length: 112bp). Thermal cycling conditions for SYBR Green (Fermentas, Sweden) consisted of a denaturation step at 95°C for 10 min followed by 40 cycles of 95°C for 15 s and 60°C for 60 s. Gene expression was analyzed using a 7900 HT real-time PCR instrument (Applied Biosystems, UK). The obtained Ct values were normalized against *GAPDH*. The primer sequences for *GAPDH* are as follows: forward sequence: GTCTCCTCTGACTTCAACA GCG; reverse sequence: ACCACCCTGTTGCTGTAGCCAA (product length:131bp). All primer sets were specific for their products (Eurofins, Ebersberg, Germany). Relative quantification of gene-expression was determined by using the ΔΔCt method. The ΔCt was calculated by subtracting the Ct of *GAPDH* from the Ct of *CXCL8* for each sample. The ΔΔCt was calculated by subtracting the ΔCt of the control sample from the ΔCt of each treated sample. Fold change was generated by using the equation 2^ΔΔCt^.

### CXCL8 degradation assay

The cell-free culture supernatants from THP-1 cells treated with heat-killed W50 were filtered using 0.2 μm filter to ensure the supernatants were free of bacterial contamination. The supernatant was diluted 10 times using RPMI1640. Several 500 μl aliquots were made and the tubes were incubated with viable or heat killed ATCC 33277 or W50 or viable E8 and K1A for 4 hours at 37°C, 5% CO_2_. Three serially diluted concentrations (10^7^ -10^9^ CFU/ml) of viable and heat killed bacteria were used. After 4 hour incubation, the samples were centrifuged at 9300 x g for 10 minutes at room temperature and the supernatant was transferred to a fresh tube and stored at -80°C until further analysis using Human CXCL8 ELISA kit (Biolegend, Sandiego, USA).

### Endotoxin quantification

The cell culture supernatants of THP-1 cells treated with different strains of bacteria were centrifuged at 9300 × g to get rid of cells, filtered through a 0.2 μm filter to eliminate bacteria and stored at -80°C until further quantified for endotoxin levels using LAL Chromogenic Endotoxin Quantitation Kit, according to manufacturer’s instructions. (Thermoscientific fisher, Sweden).

### Gingipain quantification

The cell culture supernatants of THP-1 cells treated with different strains of bacteria were collected and stored at -80°C until further quantified for argine and lysine gingipains using arginine and lysine substrates (Peptanova, Sandhausen, Germany). The arginine gingipain substrate peptide sequence is Boc-Phe-Ser -Arg-AMC (t-Butyloxycarbonyl- L-phenylalanyl- L-seryl- L-arginine- 4-methylcoumaryl- 7-amide) and the lysine gingipain substrate peptide sequence is Z-His-Glu-Lys-AMC (Benzyloxycarbonyl- L-histidyl- L-glutamyl- L-lysine- 4-methylcoumaryl-7-amide). The supernatants (200μl) were incubated with either of the substrates at a final concentration of 100μM for one hour at 37°C and the enzyme activity was read in a fluorescence microplate reader (Fluostar Optima, Ortenberg, Germany) at excitation/emission wavelength settings of 380/ 460 nm.

### Scanning laser confocal microscopy

Fluorescein isothiocyanate (FITC, Sigma Aldrich, Germany) labeled *P. gingivalis* was used to stimulate THP-1 cells (100 MOI) for 24 hours. The cells were then fixed using 4% ice-cold paraformaldehyde for 30 minutes and F-actin visualization was enabled by staining with rhodamine phalloidin (Invitrogen, Stockholm, Sweden). Nucleus was stained using 4', 6-diamidino-2-phenylindole (DAPI; Sigma Aldrich, Seelze, Germany). THP-1 cell and *P. gingivalis* interaction was analyzed by confocal microscopy (Olympus Fluoview, Hamburg, Germany).

### Statistical Analyses

The statistically significant differences of data between groups and over time, obtained from real-time qPCR, ELISA, LPS quantification and CXCL8 degradation assay were assessed using one-way ANOVA with Bonferroni post-hoc corrections. All data were represented as mean values with standard deviation. P-value < 0.05 was considered significant.

## Results

### *Porphyromonas gingivalis* induces CXCL8 production in THP-1 cells

**Figure 1:**
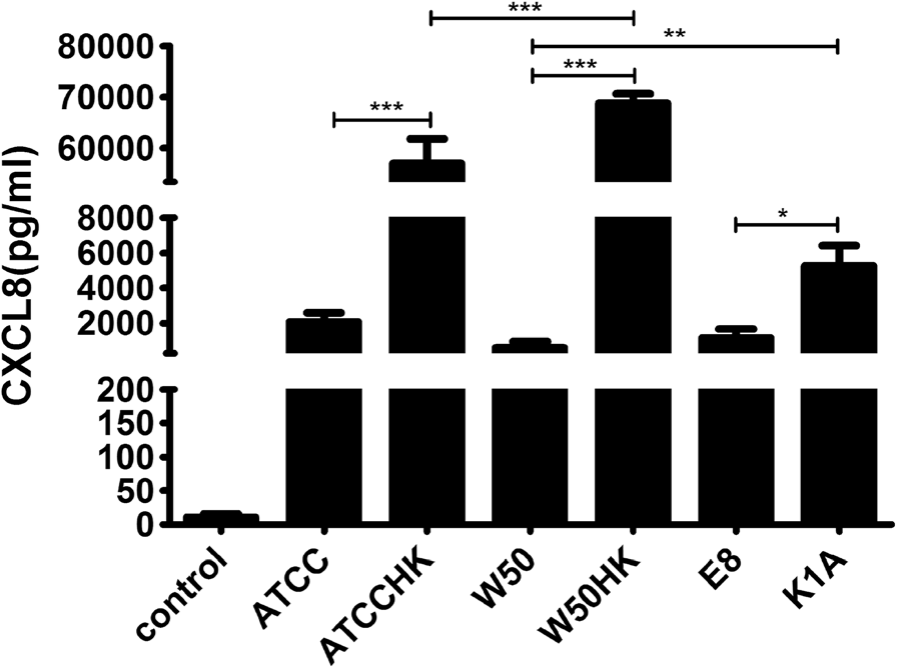
**The release of CXCL8 from THP-1 cells stimulated with wild-type or gingipain mutants of *P. gingivalis*.** THP-1 cells were stimulated with viable ATCC3277 (ATCC), W50, E8, K1A or heat-killed ATCC 33277 (ATCCHK) or W50 (W50HK) at MOI 100 for 24 hours. Values represent the mean ± SD. Statistically significant differences between the groups were determined using one-way ANOVA and Bonferroni post-hoc test. (*p < 0.05; **p < 0.01; ***p < 0.001; n = 4).

THP-1 cells were stimulated with viable or heat-killed *P. gingivalis* at a MOI 100 for 24 hours. The cell-culture supernatants were collected and quantified for CXCL8 by ELISA. The results show a marked increase in the levels of CXCL8 (2000–70,000 pg/ml) in *P. gingivalis* stimulated cells in comparison to unstimulated cells that serve as control. The highest amounts of CXCL8 were observed in the group treated with heat-killed W50 (72,000 pg/ml) and ATCC 33277 (65,000 pg/ml) respectively and this was 30 times higher compared to the levels in cells treated with viable ATCC 33277 and W50 strains. Among viable W50 and its isogenic mutants, K1A induced a significantly higher level of CXCL8 compared to W50 (p < 0.01), whereas E8 followed the same trend as viable W50 and ATCC 33277 (Figure 1) and was significantly lower than K1A (p < 0.05).

### *Porphyromonas gingivalis* markedly up-regulates *CXCL8* in THP-1 cells

**Figure 2:**
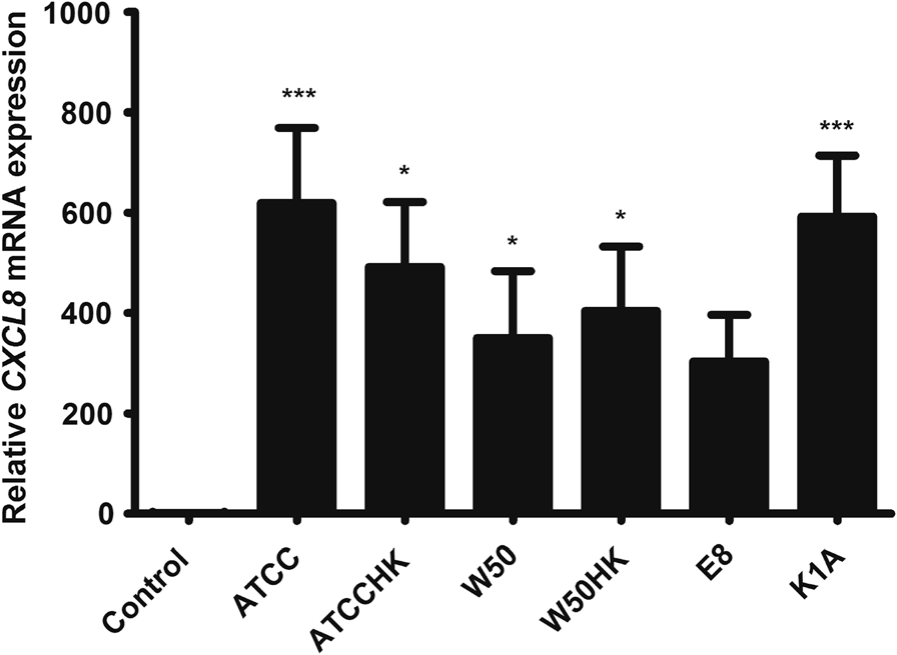
**The mRNA expression of *CXCL8 *in THP-1 cells stimulated with wild-type or gingipain mutants of *P. gingivalis*.***CXCL8* transcription following 24 hour treatment of THP-1 cells with (MOI 100) viable ATCC3277 (ATCC), W50, E8, K1A or heat-killed ATCC 33277 (ATCCHK) or W50 (W50HK). Values represent the mean ± SD. Statistically significant differences compared to the negative control. (*p < 0.05; **p < 0.01; ***p < 0.001; n = 3).

THP-1 cells were stimulated with *P. gingivalis* for 24 hours and mRNA was collected and assessed by a quantitative real-time PCR. The results were normalized to the *GAPDH* gene and the *CXCL8* gene regulation was estimated in relation to the control. The expression of *CXCL8* gene was significantly upregulated by all strains of *P. gingivalis* except for E8 (Figure 2). The *CXCL8* gene was accentuated to almost 800-fold in the groups treated with ATCC 33277 and K1A strains (p < 0.001).

### *Porphyromonas gingivalis* lipopolysaccharide and gingipains are released during incubation with *P. gingivalis*

**Figure 3:**
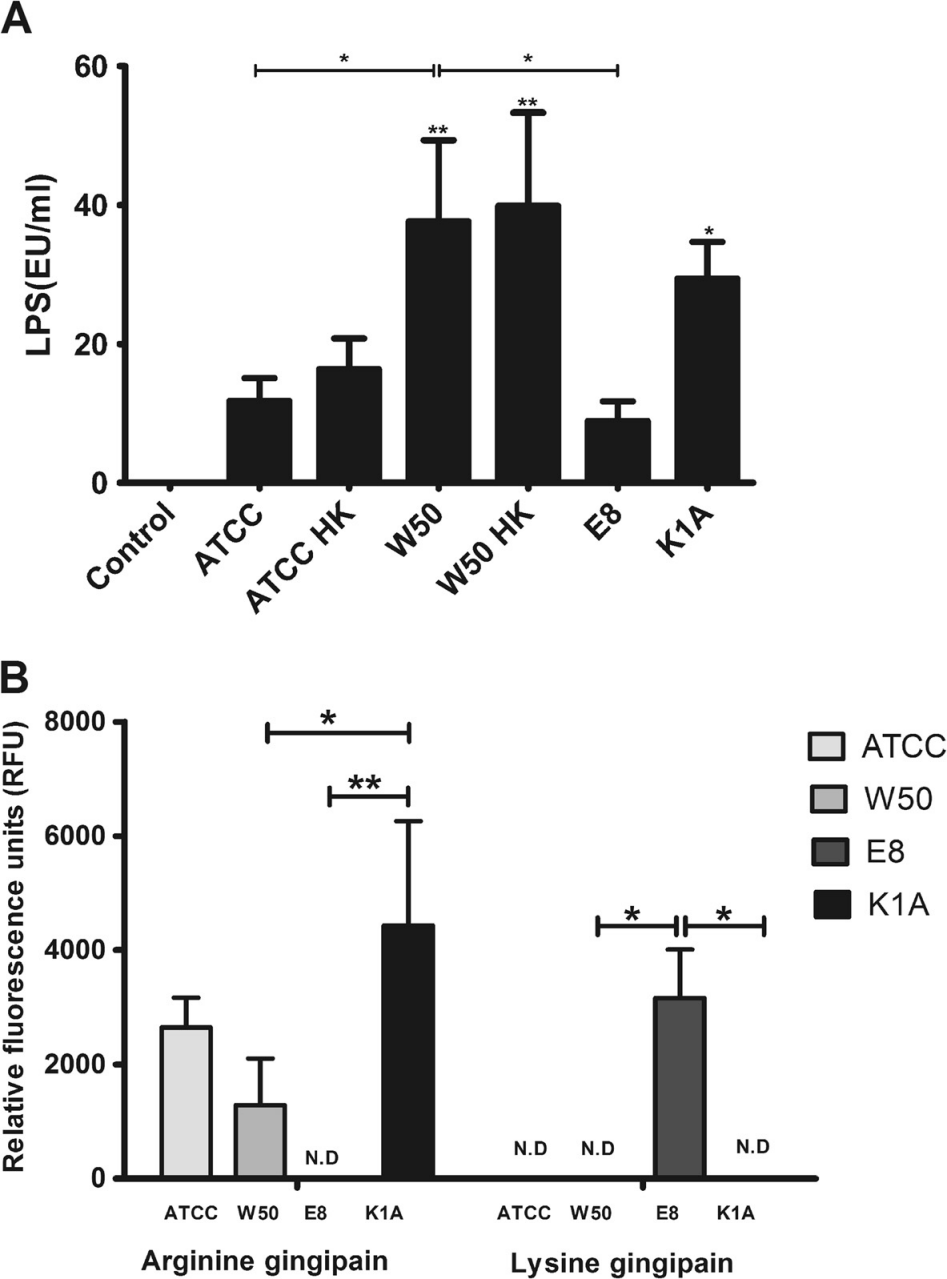
**LPS and gingipains are released from *P. gingivalis*. (A)**. The release of LPS from THP-1 cells stimulated with *P. gingivalis* (MOI 100). Significant accumulation of LPS was observed in culture supernatants of THP-1 triggered by W50, W50HK and K1A in relation to the negative control. **(B)**. Relative concentration of argine and lysine gingipains present in the culture supernatants after 24 hour stimulation of THP-1 cells with MOI 100 viable ATCC33277 (ATCC), W50, E8 or K1A (N.D: not detected). The differnces between the different stimulations were analyzed by one-way ANOVA. Values represent the mean ± SD. (*p < 0.05; **p < 0.01; ***p < 0.001; n = 3).

LPS is an important virulence factor that is capable of stimulating cells in culture [23] and hence the immunogenic nature of LPS cannot be overruled. During the co-incubation of THP-1 cells with *P. gingivalis,* there is release of LPS in to the surrounding media. No LPS could be detected in the control as expected. Significant differences were observed when comparing K1A (p < 0.05), viable and heat-killed W50 (p < 0.01) with the control. The LPS obtained on stimulating THP-1 cells with E8 (p < 0.05) was least among the different stimulations and was significantly lower compared to W50 (Figure 3A).The presence of gingipains in the culture supernatant plays an equally vital role as LPS in regulating CXCL8. As expected, supernatants from E8 stimulated THP-1 cells selectively expressed only Kgp activity (Figure 3B). Likewise, K1A stimulated THP-1 cells expressed only Rgp activity. However, in the ATCC33277 and W50 stimulated monocyte suspensions, we detected Rgp activity in the culture supernatants and there were significant differences between K1A and the wild-type strain (p < 0.05) stimulated monocyte suspensions. We could not detect Kgp activity in the W50 or ATCC33277 stimulated THP-1 cell co-cultures.

### Proteolytic efficiency of *Porphyromonas gingivalis* is mainly dependent on arginine gingipain

**Figure 4:**
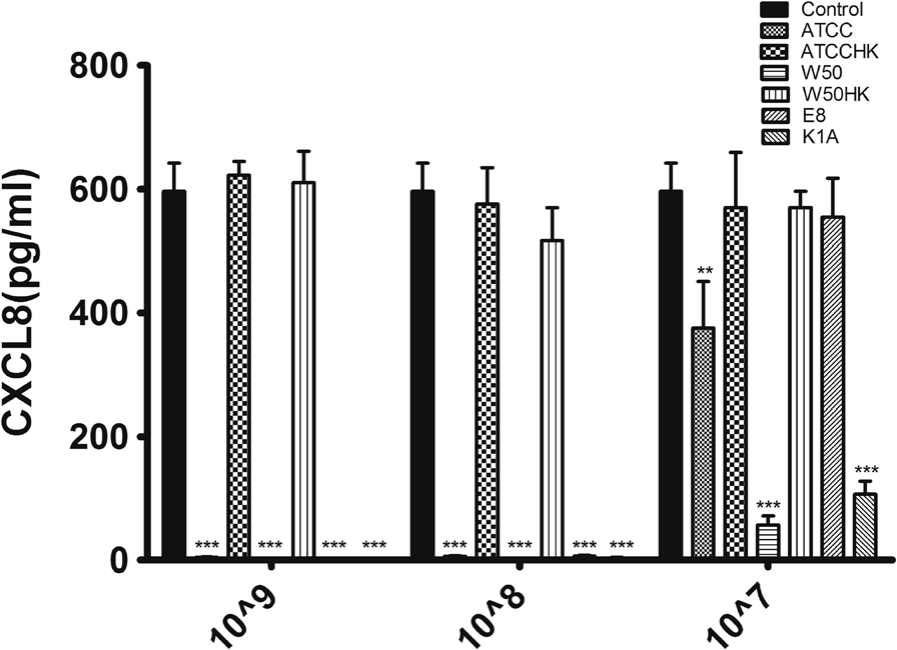
***P. gingivalis *degrades CXCL8 through gingipains.** The culture supernatant of heat-killed W50 (MOI 100) treated THP-1 cells for 24 hours was filtered and incubated with 10^7^/ml, 10^8^/ml, 10^9^/ml viable ATCC3277 (ATCC), W50, E8, K1A or heat-killed ATCC 33277 (ATCCHK) or W50 (W50HK) for 4 hours and CXCL8 was quantified by ELISA. Significant differences among each group were analyzed in relation to the control and statistics were done using one-way ANOVA. Values represent the mean ± SD. (*p < 0.05; **p < 0.01; ***p < 0.001; n = 3).

Sterile-filtered supernatants from THP-1 cells stimulated with heat-killed W50 were incubated with various strains of *P. gingivalis* in three different concentrations for 4 hours to determine CXCL8 cleaving efficiency. All of the viable strains of the two highest concentrations (10^9^ and 10^8^ CFU /ml) completely cleaved CXCL8, whereas the heat-killed strains showed no signs of proteolysis irrespective of strain or concentrations. However, the lowest concentration tested i.e. 10^7^ CFU/ml actually demonstrated that arginine gingipain (K1A) expresses highest proteolytic activity compared to lysine gingipain (E8) (Figure 4).

### Variable capacity of *P. gingivalis* for adhesion and invasion

**Figure 5:**
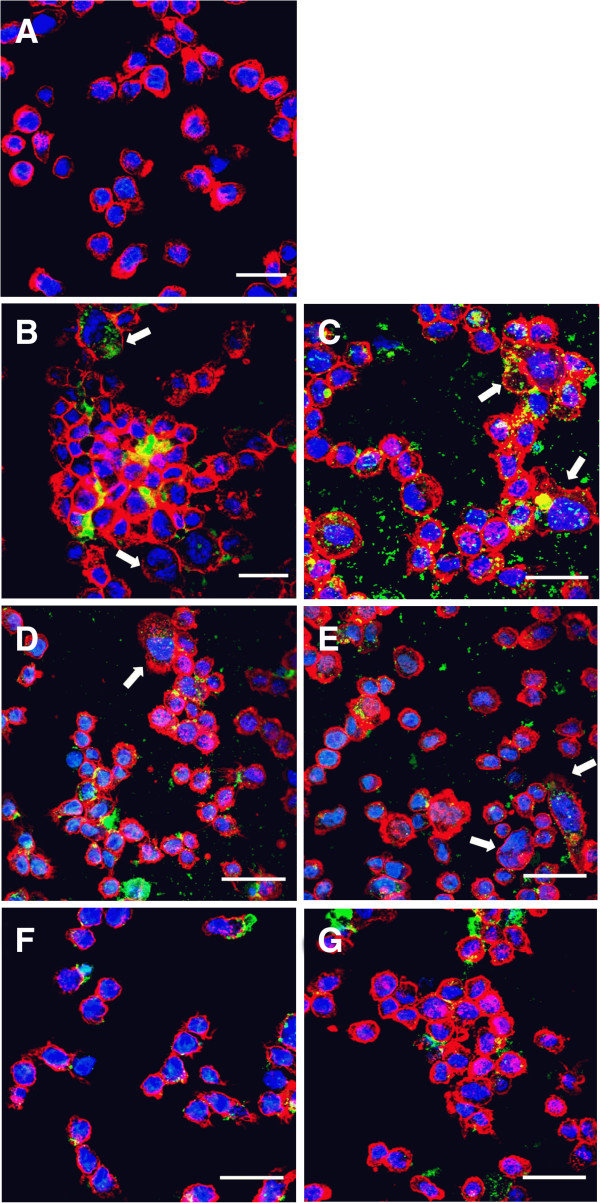
**THP-1 cells were incubated with different strains and mutants of FITC-labelled (green) *P. gingivalis *(MOI 100) for 24 hours and then stained for F-actin with rhodamine phalloidin(red) and nucleus with DAPI (blue), followed by confocal fluorosence microscopy.** Confocal images show **(A)** Control (untreated) THP-1 cells **(B)** ATCC 33277 **(C)** ATCC 33277 heat-killed **(D)** W50 **(E)** W50 heat-killed **(F)** E8 and **(G)** K1A adhere to and are found within monocytes. Large, multi-nucleated cells are indicated by white arrows in figures B, C, D and E. Bar = 40μm. 60X objective, oil immersion. Representative images from three independent experiments are shown.

Confocal images of THP-1 cells incubated with *P. gingivalis* for 24 hours shows that the different strains of the bacteria adhere to or are inside the THP-1 cells (Figures 5 A-G). An antibiotic protection assay after four hour incubation enabled us to retrieve viable *P. gingivalis* (ATCC, W50, E8 and K1A) from the cells (data not shown). THP-1 cells incubated with viable *P. gingivalis* (ATCC, W50 and K1A) have a tendency to induce morphological changes and formation of large and mixed bacteria/cell aggregates. Stimulation with the heat-killed wild-type strains resulted in larger cells and some of the infected cells were multi-nucleated. However, THP-1 cells stimulated with E8 remained mostly in suspension and were morphologically similar to the untreated cells.

## Discussion

*Porphyromonas gingivalis* is a key periodontopathogen that can seriously debilitate the immune responses and the low grade periodontal infection continuously feeds more bacteria into the blood stream [24]. The bacterial interaction with monocytes through TLRs and PARs enables a signalling cascade leading to a release of a plethora of pro-inflammatory mediators e.g. CXCL8, which is a potent chemokine responsible for neutrophil recruitment [25]. The aim of this study was to elucidate the mechanisms of *P. gingivalis* interaction with monocytes in terms of induction and expression of CXCL8. By using gingipain mutants, we found that Rgp and Kgp help *P. gingivalis* evade the immune system both in an antagonistic and synergistic manner. The results are consistent with the previous studies and would be a rational to direct future studies using peripheral blood monocytes.

Both viable and heat-killed W50 strains of *P. gingivalis* shed significant amounts of LPS during interaction with THP-1 cells and induced release of CXCL8 indicating that the TLRs are involved as LPS is heat-stable and is an agonist for TLR2 activation. We observed that the levels of CXCL8 are 30–40 times higher in THP-1 cells stimulated with heat-killed ATCC and W50 compared to cells exposed to viable bacteria. TLR2 has previously been shown to be activated by both viable and heat-killed *P. gingivalis *[26]. If the bacteria-induced release of CXCL8 is based only on LPS, there would have been no difference in CXCL8 response between viable and heat- killed bacteria, however, this was not the case.

Gingipains are trypsin-like cysteine proteases that can cleave the proteins at arginine and lysine specific sites [27]. We observed that the different strains of *P. gingivalis* (viable or heat-killed) upregulated *CXCL8* gene several hundred-folds. Uehara and colleagues investigated the possible synergistic effects of PARs, TLRs and NODs on CXCL8 production in THP-1 cells using synthetic peptide ligands and showed that gingipains stimulate the secretion of cytokines from monocytic cells through the activation of PARs with synergistic effects by PRRs [20]. Consequently, the inflammatory response induced by gingipains was not exclusively dependent on their catalytic activity since heat-inactivated bacterial preparations were still effective whereas proteolytic activity was absent by denaturation [28]. The loss of protease activity on heat-treatment was validated by a cytokine degradation functional assay and this also explains the reduced levels of extracellular CXCL8 in THP-1 cells stimulated with viable bacteria. Measuring the gingipain activity in the supernatant served as a functional validation of the isogenic gingipain mutants as well as the relative expression of arginine and lysine gingipains by the wild-type strains.

Aduse-Opoku and colleagues showed that Rgp and Kgp are secreted independent of each other [29]. It is possible that ATCC33277 and W50 could be selectively inclined to secreting Rgp in aerobic cell culture conditions. It is evident that the gene transcription patterns do not follow the protein levels and could imply that the post-secretory CXCL8 protein is being degraded by gingipains. The inflammatory character of fimbriae and bacterial DNA cannot be disregarded. Thermal inactivation of microorganisms could release bacterial DNA due to possible membrane rupture activating TLR9 and *P. gingivalis* fimbriae are also documented to activate TLR2 and aid in CXCL8 release in human aortic endothelial cells [30,31].

We show that W50 cleaves CXCL8 with the same efficiency as K1A, while ATCC 33277 is less effective. It is clear that there is variation in protease activity among the various wild-type strains of *P. gingivalis*. The degradation assay shows that at high titres, both Rgp and Kgp were equally effective in inactivating CXCL8, whereas at moderate and low titres, Rgp was more effective at degrading CXCL8. We have previously shown that CXCL8 hydrolysis can be salvaged by using leupeptin which is an arginine-specific gingipain inhibitor [19]. However, all cytokines are not cleaved alike. Stathopolou and colleagues have shown that the rate of IL-1β degradation is lower compared to that of IL-6 or CXCL8, which could be due to the primary, secondary and tertiary molecular structure of IL-1β which makes it relatively resistant to hydrolysis by Kgp. It has been shown that interleukin-6 is rapidly cleaved by both gingipains, although Kgp was found to be more effective [16,32].

The degradation assay makes it evident that Rgp is a more efficient protease at cleaving CXCL8, however, *P. gingivalis* expressing Rgp markedly amplifies CXCL8 transcription and significantly higher level of CXCL8 was present in culture supernatants of THP-1 treated with *P. gingivalis* expressing only Rgp (K1A). This could be due to a complex balance and interaction between induction and degradation. Induction of the *CXCL8* gene is due to LPS, fimbriae, bacterial outer membrane proteins, DNA and gingipains, whereas degradation of CXCL8 is only protease dependent. In addition, it has been shown that CXCL8 is a secondary cytokine, i.e. its release could also be mediated by primary cytokines, like IL-1β and TNF in an autocrine manner [33]. Hence, it is obvious that CXCL8 regulation during host pathogen interaction is very elaborate and the *in vivo* conditions are more dynamic compared to the situation *in vitro.* Also, gram negative bacteria can shed LPS in to the surrounding during cell death, outer membrane vesicle biogenesis and stress [34-36].

Confocal fluorescent images showed that THP-1 cells form aggregates when treated with *P. gingivalis*. Besides the changes in the actin cytoskeleton, *P. gingivalis* probably increased the expression of cell adhesion molecules in monocytes leading to cell aggregation [37]. THP-1cells stimulated with Kgp-deficient K1A appear to be more differentiated and aggregated, compared to the E8 (lacks Rgp)-infected THP-1 cells, which could be suggestive of a role exhibited by Rgps in modulating THP-1 cell aggregation and adhesion. Hashizume and colleagues showed that cytoadherence between THP-1 cells and endothelial cells were enhanced by treatment with *P. gingivalis *[38]. *P. gingivalis* invades or is internalized within the cells. We were able to culture intracellular bacteria by the antibiotic protection assay after 4 hour incubation showing that *P. gingivalis* survives intracellularly. The ability of ATCC 33277 to invade endothelial cells has previously been demonstrated in our lab [39]. Fimbriae possibly contributes to the intracellular presence of the ATCC strain and even sparsely fimbriated strains successfully invaded although in varying degrees indicating there could be other factors supporting adhesion and infection of cells [40]. W50 and its mutants possess type IV fimA and previous studies have confirmed that the precursor fimA can only become mature by the action of Rgp and, consequently, the lack of Rgp in E8 likely renders fimA ineffective [29,41]*.* The giant multi-nucleated cells that formed on interaction of THP-1 cells with viable or heat-killed ATCC 33277 and W50 is of additional interest and one could speculate that these giant cells could be of osteoclastic phenotype of importance in the pathogenesis of periodontitis and needs further clarification.

## Conclusion

In conclusion, our results demonstrate that both arginine and lysine gingipains could play a vital role in *P. gingivalis*-mediated immunomodulation and that Rgp appears to be cruciual in the context of CXCL8 regulation even at low concentrations. This study establishes that gingipains are capable of post-secretory chemokine paralysis which would most likely result in abberant neutrophil recruitment to the site of infection and ensuring that a low grade infection would continue unhampered. The invasion and survival of *P. gingivalis* within the monocytes could account for the safe passage of the bacteria from the site of periodontal infection to other inflamed sites. Periodontal infections are independent risk factors for several chronic systemic illness and persistent recurrent bacteremia due to denuded oral epithelium results in systemic dissemination of periodontopathogens via the vascular compartment.

## Competing interests

The authors declare that they have no competing interests.

## Author’s contributions

TB, HK and KJ design the study, KJ conducted the experiments. KJ, HK and TB analyzed the data. KJ, HK and TB drafted the article. All authors read and approved the final manuscript.
